# Persistence of cervical high-risk human papillomavirus in women living with HIV in Denmark – the SHADE

**DOI:** 10.1186/s12879-019-4377-5

**Published:** 2019-08-22

**Authors:** Kristina Thorsteinsson, Steen Ladelund, Merete Storgaard, Terese L. Katzenstein, Isik Somuncu Johansen, Gitte Pedersen, Frederikke Falkencrone Rönsholt, Lars Nørregård Nielsen, Lisbeth Nilas, Maria Franzmann, Niels Obel, Anne-Mette Lebech, Jesper Bonde

**Affiliations:** 1 Department of Infectious Diseases, Hvidovre Hospital, Kettegaards Allé 30, 2650 Hvidovre, Denmark; 2 Clinical Research Center, Copenhagen University Hospital, Hvidovre, Denmark; 3 Department of Infectious Diseases, Aarhus University Hospital, Skejby, Denmark; 4 Department of Infectious Diseases, Copenhagen University Hospital, Rigshospitalet, Copenhagen, Denmark; 5 Institute of Clinical Medicine, University of Copenhagen, Copenhagen, Denmark; 6 Department of Infectious Diseases, Odense University Hospital, Odense, Denmark; 7 Department of Infectious Diseases, Aalborg University Hospital, Aalborg, Denmark; 8 Department of Infectious Diseases, Nordsjællands Hospital, Hillerød, Denmark; 9 Department of Obstetrics and Gynaecology, Copenhagen University Hospital, Hvidovre, Denmark; 10 Molecular Pathology Laboratory, Department of Pathology, Copenhagen University Hospital, Hvidovre, Denmark

**Keywords:** Women living with HIV, Immunodeficiency, HPV persistence, High-risk HPV, HPV genotype distribution, Cervical cancer

## Abstract

**Background:**

Women living with HIV (WLWH) have high rates of persistent high-risk human papillomavirus (hrHPV) infections and cervical cancer. We aimed to assess the distribution of hrHPV genotypes, risk factors of type-specific hrHPV persistence, and high-grade squamous intraepithelial lesions or worse (≥HSIL) in WLWH in Denmark.

**Methods:**

From the prospective Study on HIV, cervical Abnormalities and infections in women in Denmark (SHADE) we identified WLWH with a positive hrHPV test during the study period; 2011–2014. HIV demographics were retrieved from the Danish HIV Cohort Study and pathology results from the The Danish Pathology Data Bank. Logistic regression was used to identify risk factors associated with persistent hrHPV infection (positivity of the same hrHPV type in two samples one-two years after the first hrHPV positive date) and ≥ HSIL.

**Results:**

Of 71 WLWH, 31 (43.7%) had persistent hrHPV infection. Predominant hrHPV genotypes were HPV58, 52, 51, and 35 and most frequently observed persistent genotypes were HPV52, 33 and 31. CD4 < 350 cells/μL predicted genotype-specific hrHPV persistence (adjusted OR 4.36 (95%CI: 1.18–16.04)) and ≥ HSIL was predicted by prior AIDS (adjusted OR 8.55 (95% CI 1.21–60.28)).

**Conclusions:**

This prospective cohort study of well-treated WLWH in Denmark found a high rate of persistent hrHPV infections with predominantly non-16/18 hrHPV genotypes. CD4 count < 350 cells/μL predicted hrHPV persistence, while prior AIDS predicted ≥HSIL.

**Electronic supplementary material:**

The online version of this article (10.1186/s12879-019-4377-5) contains supplementary material, which is available to authorized users.

## Highlights

Persistent hrHPV infection with non-16/18 was highly prevalent in women living with HIV.

Multiple hrHPV infections was seen in a third of participating women living with HIV.

Low CD4 count predicted hrHPV persistence.

Cytological abnormalities were predicted by short duration of ART and prior AIDS.

## Background

Persistent infection with high-risk human papillomavirus (hrHPV) is a prerequisite for the development of cervical cancer (CC) [1]. Yet, the majority of acquired HPV infections are transient and naturally cleared by the immune system within a few months [1]. HrHPV genotypes differ in their carcinogenic potential and therefore genotype distribution has an impact on the risk of CC development [1–5].

Two modalities are available to prevent CC; primary prevention through vaccine, and secondary prevention through screening with cytology and hrHPV testing. As current vaccines do not cover all oncogenic HPV genotypes, nor eliminate existing infections at time of vaccination [6], screening remains an important preventive effort. In the general population, the impact of cervical screening on CC mortality is well described [7]. However, the inability of current screening technologies to distinguish between transient and persistent HPV infection is an important cause of overtreatment [8].

Women living with HIV (WLWH) have a higher prevalence of hrHPV, a higher rate of persistent hrHPV infection, and a higher rate of cervical intraepithelial neoplasia (CIN) and CC compared to HIV-negative women [1, 4, 9–12]. Moreover, the hrHPV genotype distribution differs between the two populations [13–16].

With the longevity gained from combined antiretroviral therapy (ART) [17, 18] the burden of HPV-related cancer is of increasing concern in people living with HIV (PLWH) [17]. Moreover, the impact of ART on hrHPV and cervical lesions are still debatable [9]. As primary prevention, European HIV guidelines recommend HPV vaccination of WLWH through age 26 [19]. As secondary prevention, HPV based screening is currently substituting cytology screening in many European countries. Yet, the high hrHPV prevalence amongst WLWH challenges HPV based screening as this results in lower specificity compared to cervical cytology screening [20]. The ideal way to adopt screening programs for WLWH is therefore still unresolved.

Data on hrHPV persistence in WLWH is scarce and heterogeneous, therefore the aim of this study was to assess the distribution of hrHPV genotypes, risk factors of type-specific hrHPV persistence, cervical cytological abnormalities and CIN in WLWH in Denmark.

## Methods

### Setting

In Denmark, the estimated HIV prevalence among adults is 0.1% [21]. Medical care, including ART, is tax-paid and provided free-of-charge [13]. HIV treatment is restricted to nine specialized medical centers with outpatient visits every 3–6 months [22]. Six of these centers participated in this prospective, observational cohort study of WLWH in Denmark [23] called Study on HIV, cervical Abnormalities and infections in women in Denmark (SHADE) [13, 23].

In the SHADE, WLWH were consecutively enrolled from 1 February 2011 to 1 February 2012 and followed-up after 6, 12 and 24 months. Inclusion criteria were HIV-1 infection and ≥ 18 years of age. At each visit all participants underwent a gynecological examination including an HPV test and a cervical cytology sample. In the present study, WLWH entered the analyses the day they were diagnosed hrHPV positive. If applicable, WLWH were censured at time of conization/hysterectomy. Exclusion criteria were pregnancy at first visit, prior hysterectomy, alcohol and/or drug abuse impeding adherence to the protocol. Information on tobacco use, age at sexual debut, lifetime sexual partners, prior condyloma, HPV vaccination status, and contraceptive use was obtained from a questionnaire (Additional file 1) [13, 23]. The EpiData Entry program was used for double manual data entry [24].

### Registry data

#### Civil registration system (CRS)

The CRS is a national registry of all Danish residents [25]. A 10-digit personal identification number (PIN) is assigned to each individual at birth or immigration. This PIN was used to link data from the SHADE cohort, the Danish HIV Cohort Study (DHCS) and the The Danish Pathology Data Bank (DPDB).

#### Danish HIV cohort study (DHCS)

The DHCS is a prospective, observational, nationwide, multicenter cohort study of all PLWH seen at the Danish HIV centers since 1 January 1995 and has been described in detail elsewhere [22]. We retrieved HIV demographics from the DHCS.

#### The Danish pathology data Bank (DPDB)

The DPDB contains nationwide records of all pathology specimens analyzed in Denmark since 1997 [26]. Cytology and histology samples were retrieved using the Systemized Nomenclature of Medicine (SNOMED) code of cervix uteri and uterus: T8x2*, T8x3*, T82* and T83*.

### Cytology and HPV testing

Cervical samples were collected using the combi brush (Rovers, Oss, The Netherlands) in SurePath liquid based cytology media (BD Diagnostics, Durham, NC, US) and analyzed at the Department of Pathology, Copenhagen University Hospital, Hvidovre. The Bethesda 2001 system was used to report the cytology results [27]. Samples were classified as normal, atypical cells of undetermined significance (ASCUS), low-grade squamous intraepithelial lesions (LSIL), or high-grade squamous intraepithelial lesions (HSIL) (including atypical squamous cells - cannot exclude HSIL (ASC-H), atypical glandular cells and adenocarcinoma in situ), squamous cell-and adenocarcinoma. Histology reports of CIN grade 1 (CIN1), CIN grade 2 (CIN2), CIN grade 3 (CIN3) and CC were based on biopsies. Cytology and histology results were presented as the worst cytology/histology result diagnosed concurrent with or after a positive hrHPV test.

Cervical samples for HPV testing were collected using flocked swabs (UTM-RT viral transport media Flocked Polyester Swabs, Copan Diagnostics, Inc., Murrieta, CA). All samples were stored at room temperature and examined by the CLART HPV2 assay (Genomica, Madrid, Spain) at the Department of Pathology, Copenhagen University Hospital, Hvidovre. PCR amplification of genotype specific HPV L1 fragments from 35 individual HPV genotypes was performed; of these 13 were hrHPV genotypes: HPV16, 18, 31, 33, 35, 39, 45, 51, 52, 56, 58, 59, and 68 [28].

### Definition of type-specific persistence versus clearance

Type-specific persistence was defined as positivity of the same hrHPV type in two separate cervical samples having been taken at least 1 year and at most 2 years after the date the patient was first hrHPV positive. Clearance was defined as having one or more negative results after a hrHPV positive sample [29]. WLWH with type-specific persistence of one genotype and clearance of another were reported as having persistent HPV infection.

### Statistical analysis

Continuous variables were summarized as median and interquartile ranges (IQR) and compared using the Wilcoxon rank sum test. Categorical variables were reported as counts and percentages and compared using the chi-square test or Fisher’s exact test, where appropriate. Univariate and multiple logistic regression analyses were used to identify predictors of persistent hrHPV, ASCUS or worse (≥ASCUS), LSIL or worse (≥LSIL), and HSIL or worse (≥HSIL) expressed as odds ratios (OR) and 95% confidence intervals (CI). We chose six candidate predictor variables a priori due to current knowledge on risk factors of HPV infection [13, 30, 31]; age (18–34 vs. ≥ 35 years), race (White, Asian, and Black), duration of ART (years on ART), AIDS prior to inclusion, smoking status (never smoker vs. current smoker/ex-smoker) and latest CD4 count when first hrHPV positive (< 350 and ≥ 350 cells/μL). Predictors of ≥ASCUS, ≥LSIL, and ≥ HSIL were estimated by including the aforementioned variables and additionally persistent hrHPV infection. Since duration of ART, prior AIDS and the CD4 count are dependent covariates, two models were used: 1) a model where all variables but the latest CD4 count was included and 2) a model where duration of ART and prior AIDS were replaced by latest CD4. From the second model only, the OR of the CD4 count was presented.

In all analyses *p*-values < 0.05 (two-tailed) were considered significant. For category variables with more than two outcome categories (df > 1), we controlled for repeated testing by estimating the combined *p*-value. We excluded individuals with missing explanatory values from the multiple regression analyses. The validity of the model was tested using the Hosmer and Lemeshow Goodness-of-Fit Test. SAS statistical software version 9.4 (SAS Institute Inc., Cary, NC, USA) was used for data analysis.

## Results

**Fig. 1:**
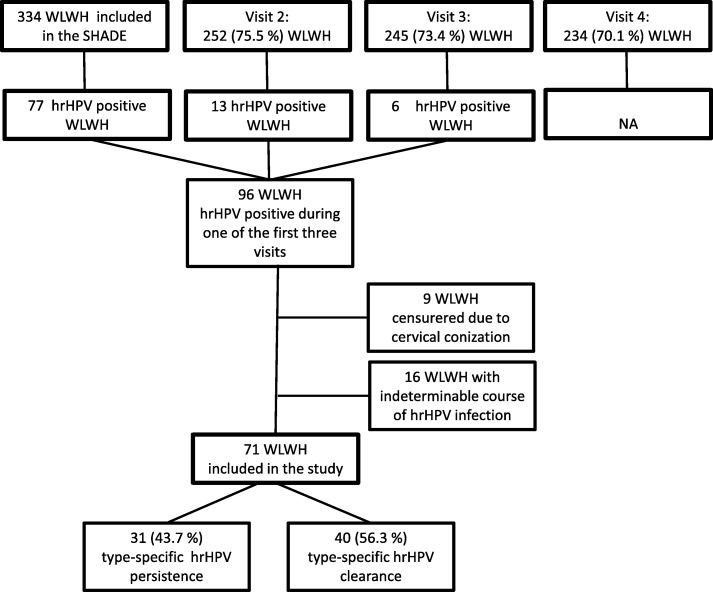
Flowchart of inclusion, follow-up and course of hrHPV infection. WLWH = Women living with HIV. SHADE = Study on HIV, cervical Abnormalities and infections in women in Denmark. hrHPV = High-risk human papillomavirus

Overall, 334 WLWH were included in the overall SHADE study, and of these, 252 (75.5%), 245 (73.4%) and 234 (70.1%), respectively, participated at the subsequent visits at 6, 12 and 24 months after inclusion (Fig. 1). During follow-up, 96 WLWH (28.7%) presented with at least one hrHPV-positive test through the course of three visits. Of these, 25 WLWH (26.0%) had an indeterminable course of HPV infection either if no follow-up tests were performed or censored at time of conization (none were hysterectomized). In total, 71 WLWH were included in this analysis (Fig. 1).

Of the 71 included WLWH, 31 (43.7%) had persistent hrHPV infection (Fig. 1). Table 1 shows baseline characteristics of WLWH with persistent and cleared hrHPV infection. Median age was 42.5 (IQR 33.8–49.5) years. The majority of WLWH (96.7 and 83.8%) were sexually infected with HIV and were on ART with HIV-RNA < 40 copies/mL (80.8 and 72.7%). More WLWH in the hrHPV persistence group had a CD4 count< 350 cells/μL (40.7% vs. 15.2%, *p* = 0.03) (Table 1).

**Table 1:** Characteristics of study participants with persistent^a^ and cleared^b^ high-risk (hr) human papillomavirus (HPV) infection during the study period 2011–2014 (*n* = 71)

	hrHPV persistence	hrHPV clearance	*p*-value
Number of individuals, n(%)	31 (43.7)	40 (56.3)	–
HIV duration (years), median (IQR)	7.6 (3.5–17.3)	11.5 (6.2–16.6)	0.45
Age at inclusion (years), median (IQR),	46.6 (34.0–51.3)	39.6 (33.5–47.4)	0.18
Race, n(%)			
White	13 (41.9)	20 (51.2)	0.29
Asian	4 (12.9)	1 (2.6)	
Black	14 (45.2)	17 (43.6)	
Other	0 (0)	1 (2.6)	
(missing)	(0)	(1)	
Place of HIV transmission, n(%)			
Denmark	11 (36.7)	16 (42.1)	0.05
Europe + US	1 (3.3)	6 (15.8)	
Africa	14 (46.7)	16 (42.1)	
Asia	4 (13.3)	0 (0)	
(missing)	(1)	(2)	
Mode of transmission, n(%)			
Heterosexual	29 (96.7)	31 (83.8)	0.32
IDU	1 (3.3)	4 (10.8)	
Other	0 (0)	2 (5.4)	
(missing)	(1)	(3)	
ART^c^ at inclusion, n(%)			
Yes	30 (96.8)	37 (92.5)	0.63
No	1 (3.2)	3 (7.5)	
(missing)	(0)	(0)	
ART^c^ duration (years), median (IQR)	5.4 (3.4–11.4)	8.1 (2.6–12.1)	0.66
On ART^c^ with HIV RNA < 40 copies/mL, n(%)			
Yes	21 (80.8)	24 (72.7)	0.47
No	5 (19.2)	9 (27.3)	
(missing)	(5)	(7)	
CD4 count at inclusion (cells/μL), n(%)			
< 350	11 (40.7)	5 (15.2)	0.026
≥ 350	16 (59.3)	28 (84.8)	
(missing)	(4)	(7)	
AIDS prior to inclusion, n(%)			
Yes	10 (32.3)	6 (15.4)	0.10
No	21 (67.7)	33 (84.6)	
(missing)	(0)	(1)	
HPV vaccination prior to inclusion, n(%)			
No	29 (96.7)	40 (100.0)	0.43
Yes (4-valent HPV vaccine)	0 (0)	0 (0)	
Yes (2-valent HPV vaccine)	0 (0)	0 (0)	
Yes (name of vaccine unknown)	1 (3.3)	0 (0)	
(missing)	(1)	(0)	
Age at sexual debut, n(%)			
< 16	10 (32.3)	14 (35.0)	0.81
≥ 16	21 (67.7)	26 (65.0)	
(missing)	(0)	(0)	
Lifetime sexual partners, n(%)			
< 5	7 (22.6)	5 (12.5)	0.59
5–14	14 (45.2)	23 (57.5)	
15–25	3 (9.7)	5 (12.5)	
> 25	7 (22.6)	7 (17.5)	
(missing)	(0)	(0)	

^a^Persistent = Type-specific persistence was defined as positivity of the same hrHPV type in two separate cervical samples having been taken at least 1 year and at most 2 years after the date the patient was first hrHPV positive, ^b^Cleared = having one or more negative results after an hrHPV positive sample, ^c^ART = antiretroviral therapy

Multiple (≥2) hrHPV infections were present in 23 of 71 women at inclusion (32.4%). Predominant hrHPV genotypes were HPV58 (*n* = 20; 28.1%), 52 (*n* = 14; 19.7%), 51 (*n* = 13; 18.3%) and 35 (n = 13; 18.3%). HrHPV genotypes most likely to persist were HPV52, 33 and 31 (78.6, 42.9, and 40.0%) (Table 2). One woman was vaccinated against HPV (name of vaccine unknown) (Table 1) and had persistent HPV35.

**Table 2:** Distribution of persistent^a^ and cleared^b^ high-risk (hr) human papillomavirus (HPV) infections in 71 included WLWH

	HrHPV infections, n(% of total number of WLWH) (*n* = 112)	HrHPV persistence, n(%)	HrHPV clearance, n(%)
hrHPV16	12 (10.7)	4 (33.3)	8 (66.7)
hrHPV18	5 (4.4)	0 (0)	5 (100.0)
hrHPV31	10 (8.9)	4 (40.0)	6 (60.0)
hrHPV33	7 (6.3)	3 (42.9)	4 (57.1)
hrHPV35	13 (11.6)	5 (38.5)	8 (61.5)
hrHPV39	4 (3.6)	0 (0)	4 (100.0)
hrHPV45	3 (2.7)	1 (33.3)	2 (66.7)
hrHPV51	13 (11.6)	4 (30.8)	9 (69.2)
hrHPV52	14 (12.5)	11 (78.6)	3 (21.4)
hrHPV56	5 (4.4)	1 (20.0)	4 (80.0)
hrHPV58	20 (17.9)	5 (25.0)	15 (75.0)
hrHPV59	0 (0)	0 (0)	0 (0)
hrHPV68	6 (5.4)	0 (0)	6 (100.0)

WLWH = women living with HIV

^a^Persistent = Type-specific persistence was defined as positivity of the same hrHPV type in two separate cervical samples having been taken at least 1 year and at most 2 years after the date the patient was first hrHPV positive, ^b^Cleared = having one or more negative results after an hrHPV positive sample

Predictor of HPV persistence was CD4 < 350 cells/μL (adjusted OR 4.36 (95%CI: 1.18–16.04), Table 3). Predictor of concurrent cytology findings of ≥ASCUS and ≥ LSIL was short duration of ART (≥ASCUS: adjusted OR 0.85 (95%CI: 0.74–0.99), Table 4 and Additional file 2, ≥LSIL: adjusted OR 0.83 (95%CI: 0.71–0.97), Table 4 and Additional file 3). Despite low numbers, ≥HSIL was predicted by prior AIDS (adjusted OR 8.55 (95%CI 1.21–60.28), Table 4 and Additional file 4). Due to a limited number of events predictors of CIN were not estimated. There was no significant difference between the HPV persistence and the HPV clearance group and their cytological and histological outcomes.

**Table 3:** Unadjusted and adjusted odds ratios for predictors of persistent high-risk (hr) human papillomavirus (HPV) infection (*n* = 71)

Predictors of persistence	HrHPV Persistence (*n* = 31)	HrHPV Clearance (*n* = 40)	Unadjusted odds ratios	*p*-value	Adjusted odds ratios^a, b^	*p*-value
Age when first hrHPV positive, n(%)						
≥ 35 years	22 (44.9)	27 (55.1)	1.00	–	1.00	–
18–34 years	9 (40.9)	13 (59.1)	0.85 (0.31–2.36)	0.75	0.52 (0.13–2.07)	0.35
(missing)	(0)	(0)				
Race, n(%)						
White	13 (39.4)	20 (60.6)	1.00	–	1.00	–
Asian	4 (80.0)	1 (20.0)	6.15 (0.62–61.37)	0.12	7.38 (0.68–80.17)	0.10
Black	14 (45.2)	17 (54.8)	1.27 (0.47–3.42)	0.64	1.71 (0.52–5.64)	0.38
(missing)	(0)	(2)				
Combined p-value				0.30		0.22
ART duration, (years)						
Median (IQR)	5.4 (3.3–11.4)	8.1 (2.6–12.1)	0.97 (0.88–1.07)	0.52	0.91 (0.80–1.04)	0.17
(missing)	(1)	(3)				
AIDS prior to inclusion, n(%)						
No	21 (38.9)	33 (61.1)	1.00	–	1.00	–
Yes	10 (62.5)	6 (37.5)	2.62 (0.83–8.28)	0.10	3.77 (0.99–14.38)	
(missing)	(0)	(1)				0.05
Smoking status, n(%)						
Never smoker	17 (43.6)	22 (56.4)	1.00	–	1.00	
Current smoker/ Ex-smoker	14 (43.8)	18 (56.2)	1.01 (0.39–2.58)	0.99	1.13 (0.35–3.68)	0.84
(missing)	(0)	(0)				
CD4 count when first hrHPV positive (cells/μL), n(%)						
≥ 350	16 (36.4)	28 (63.6)	1.00	–	1.00	
350	11 (68.8)	5 (31.2)	3.85 (1.13–13.07)	0.03	4.36 (1.18–16.04)	0.03
(missing)	(4)	(7)				

ART = combined antiretroviral therapy

^a^The validity of the model was tested using the Hosmer and Lemeshow Goodness-of-Fit Test, ^b^Duration of ART, AIDS prior to inclusion and CD4 count are dependent covariates and where calculated using two models: A model where all variables, but CD4 at inclusion were included and a model where duration of ART and AIDS prior to inclusion were replaced by CD4. We only present the OR of the CD4 count from the second model

**Table 4:** Worse cytological and histological findings in women living with HIV with persistent^a^ and cleared^b^ high-risk (hr) human papillomavirus (HPV) infection during the study period 2011–2014 (n = 71)

	HPV persistence (n = 31)	HPV clearance (n = 40)
Cytology		
Normal cytology, n(%)	16 (36.4)	28 (63.6)
ASCUS^c^, n(%)	0 (0)	1 (100.0)
LSIL^d^, n(%)	9 (50.0)	9 (50.0)
HSIL^e^, n(%)	6 (75.0)	2 (25.0)
Carcinoma, n(%)	0 (0)	0 (0)
Histology		
Normal histology, n(%)	3 (30.0)	7 (70.0)
CIN1^f^, n(%)	4 (66.7)	2 (33.3)
CIN2^g^, n(%)	3 (75.0)	1 (25.0)
CIN3^h^, n(%)	2 (100.0)	0 (0)
Carcinoma verified histologically, n(%)	0 (0)	0 (0)

^a^Persistent = Type-specific persistence was defined as positivity of the same hrHPV type in two separate cervical samples having been taken at least 1 year and at most 2 years after the date the patient was first hrHPV positive; ^b^Cleared = having one or more negative results after an hrHPV positive sample; ^c^*ASCUS* atypical cells of undetermined significance; ^d^LSIL = low-grade squamous intraepithelial lesions; ^e^*HSIL* high-grade squamous intraepithelial lesions including atypical squamous cells - cannot exclude HSIL (ASC-H), atypical glandular cells (AGC) and adenocarcinoma in situ (AIS); ^f^*CIN1* cervical intraepithelial neoplasia grade 1; ^g^*CIN2* cervical intraepithelial neoplasia grade 2; ^h^*CIN3* cervical intraepithelial neoplasia grade 3

## Discussion

In this prospective cohort study of well-treated, hrHPV-positive WLWH in Denmark, more than 40% of the hrHPV positive had persistent hrHPV infection, a third had multiple hrHPV infections. HrHPV genotypes other than HPV16 and 18 predominated. CD4 < 350 cells/μL predicted genotype-specific hrHPV persistence. Cytological abnormalities were predicted by short duration of ART and prior AIDS.

Some have challenged the idea of inclusion of concurrent HPV testing in WLWH, due to the high incidence of cervical hrHPV in WLWH > 30 years of age, which may reduce the benefit of a combined HPV test and cervical cytology for CC screening in this population [32]. Still, a better triage test for HPV-infected WLWH to improve efficiency of cervical screening is of importance. While HPV testing offers many advantages over liquid-based cytology and is more sensitive in detection of high-grade precancerous lesions [33, 34], cervical cytology is more specific. However, in the WLWH population with a high HPV prevalence, HPV screening can lead to over-referral and adverse events associated with overtreatment (6). In Denmark more than 10% of WLWH have had a cone biopsy performed compared with 4% of women in the general population and the intervention was performed at earlier CIN stages [35]. Whether this reflects a more aggressive clinical practice for WLWH than women without HIV is unknown.

The relationship between HIV infection, persistent hrHPV and CC is not well understood [36]. It has been suggested that a combination of increased susceptibility to HPV infection, decreased ability to clear the infection caused by impaired cell-mediated immunity, and reactivation of latent HPV infection associated with immunosuppression could explain the both increased prevalence, incidence and persistence of hrHPV among WLWH [10]. A small study of 19 WLWH and 19 HIV-negative women suggested that high regulatory T-cells and low plasmacytoid dendritic cells levels might be associated with hrHPV persistence in both groups [36].

In Denmark, CC is highly prevalent [37] and our current findings on persistence is mirrored in a study including 2874 women from the general Danish population where hrHPV persistence was found in 31.4% of the hrHPV positive women [29], with a follow-up of 1 to 4.5 years vs. 2 years in the present study.

In the present study persistent genotype specific hrHPV infection was defined as hrHPV persistence lasting at least 1 year as done previously [29] and found in 43.7% of WLWH. Comparison of hrHPV persistence rates with other studies is in general hampered by variations in HPV detection between used molecular HPV tests [30] and in the definition of persistence [1, 38]. A study in WLWH from Nigeria found a 6 months hrHPV persistence rate of 15.9% using the SPF10-LiPA HPV test, which is highly sensitive for detection of HPV genotypes [14]. Likewise, the MACH-1 study, where the persistence rate was 55.8%, defined persistence as being positive in two samples more than 6 months apart [32] using the Hybrid Capture 2 (HC2) hybridization assay and genotyping HC2 positive samples using HPV Line Blot Assay. For both studies, the interval to define persistence was short, and the used HPV testing strategy designed to pick up HPV infections with a high sensitivity. In contrast, a Brazilian study of HPV infected, pregnant WLWH with a test interval of 18 months found hrHPV persistence in 18 of 90 women (20.0%) if the study was re-calculated to the 13 genotypes considered hrHPV in the present study [30], but the pregnant status of the enrolled women changes the premise as previous studies have found HPV clearance to be more likely occur late in pregnancy or postpartum, than in the first two trimesters [30, 39]. Finally, an Italian study found 27/35 (77.1) WLWH to have hrHPV persistence at the end of 14 months follow-up [40].

Evidence is conflicting as to whether any hrHPV genotype persists longer than others [1]. Yet, it is well established that individual hrHPV genotypes differ in their relative carcinogenic potential [1–5]. Compared to HIV-negative women, as in the large Danish study [29], our and other previous reports show that WLWH have a higher proportion of multiple HPV infections [32, 41] with HPV genotypes other than HPV16 and 18 prevailing [13–16, 41]. In the SHADE study, the predominant hrHPV genotypes were HPV58, 52, 51, and 35 [13]. A worldwide review of almost 20,000 WLWH found that in the African region HPV16, 18 and 45 positivity increased consistently with severity of cervical diagnosis compared to normal cytological samples as well as in confirmed CC [4]. Among European WLWH only HPV33 positivity increased by severity [4]. The finding of non-HPV16 genotypes in high-grade CIN and CC in WLWH is proposed to be explained by the lower influence of immunodeficiency on HPV16 than on other hrHPV genotypes, due to the better intrinsic ability of HPV16 to evade immunological control even in immunocompetent individuals [42].

In an update on the natural history of HPV, Moscicki et al. [1] stated that the strongest factor resulting in persistent infection is likely the lack of an adequate immune response. This statement is supported by the results of the present and other cohorts [12, 30, 43], where current low CD4 count predicted hrHPV persistence. The impact of ART on hrHPV and HPV-related disease is still controversial [10, 11, 31]. Median ART duration in the present study was > 5 years, but no association with ART duration and hrHPV persistence was found. Since this study was performed before the publication of the START study advocating for early ART initiation [44], ART initiation was deferred until the occurrence of the following; HIV-related disease, AIDS defining illness, pregnancy and CD4 count< 350 cells/μl [45]. Therefore, WLWH initiated ART at later HIV stages than in recent studies, which is known to impact morbidity and mortality [11]. A study by Konopnicki et al. [31] suggests that > 3 years of undetectable HIV-RNA and > 1.5 years of CD4 counts of > 500 cells/μL are needed to obtain hrHPV clearance and demonstrated a decreased risk of persistent hrHPV infection in WLWH with sustained immunological reconstitution and long-lasting HIV suppression [31]. Other studies finding no effect of ART on HPV were mostly performed in the early ART era, and might therefore be affected by factors such as higher toxicity in older ART drugs, resulting in treatment delay and/or poor adherence [11]. The contradictory results regarding ART and HPV persistence might be owing to complex interactions between HPV, ART and duration and level of immunodeficiency/immune reconstitution [31, 46]. One study found that higher HPV16 viral loads were predictive of persistent HPV16 infection [47].

Our analyses of predictors of cytological abnormalities should be interpreted with caution due to the sample size. Nevertheless, ≥ASCUS and ≥ LSIL were predicted by short duration of ART, while ≥HSIL was predicted by prior AIDS, serving as a proxy of prior severe immunodeficiency. This difference in predictor may reflect that HPV infection is thought to progress via two pathways [17]; transient low-grade lesions that do not progress to high-grade CIN reflecting active HPV replication, and high-grade (precancerous) lesions mirroring HPV-induced transformation [17].

### Limitations and strengths

Strengths include the well-characterized cohort and the use of nationwide registries. Furthermore, cytological abnormalities were followed-up by histopathological confirmation. Finally, HPV and cytology analyses were performed routinely in a tested, high-throughput, quality-controlled and quality-assured laboratory. The study has limitations including the lack of a control group, the fact that some patients did not participate in all planned visits, the relatively small sample size and accordingly low rate of CIN, precluding us from performing detailed analyses on high grade CIN. Finally, detection of HPV at two-time points could reflect clearance and subsequent reinfection and not real viral persistence [48], yet this limitation can only be overcome by massive, short interval testing outside the scope of most studies.

## Conclusion

Studies on hrHPV persistence in WLWH are diverse and scarce. This prospective cohort study of well-treated WLWH in Denmark found a high rate of persistent hrHPV infections with predominantly non-16/18 hrHPV genotypes. Low CD4 count predicted hrHPV persistence, while prior AIDS predicted ≥HSIL, which supports continued focus on previously and currently immunocompromised WLWH with respect to screening for HPV-related cancers.

## Additional files

Additional file 1: Questionnaire at visit 1 (inclusion). A questionnaire regarding information on tobacco use, age at sexual debut, lifetime sexual partners, prior condyloma, HPV vaccination status, and contraceptive use. (DOC 127 kb)

Additional file 2: Unadjusted and adjusted odds ratios for predictors of atypical cells of undetermined significance or worse (ASCUS+). A table presenting the unadjusted and adjusted odds ratios for predictors of atypical cells of undetermined significance or worse (ASCUS+). (DOCX 18 kb)

Additional file 3: Unadjusted and adjusted odds ratios for predictors of low-grade squamous intraepithelial lesions or worse (LSIL+). A table presenting the unadjusted and adjusted odds ratios for predictors of low-grade squamous intraepithelial lesions or worse (LSIL+). (DOCX 17 kb)

Additional file 4: Unadjusted and adjusted odds ratios for predictors of high-grade squamous intraepithelial lesions or worse (HSIL+). A table presenting the Unadjusted and adjusted odds ratios for predictors of high-grade squamous intraepithelial lesions or worse (HSIL+). (DOCX 18 kb)

## Data Availability

The datasets generated and analysed during the current study are not publicly available due to confidentiality concerns but are available from the corresponding author on reasonable request.

## Abbreviations

ART: Combined antiretroviral therapy
ASC-H: Atypical squamous cells - cannot exclude HSIL
ASCUS: Atypical squamous cells of undetermined significance
CC: Cervical cancer
CI: Confidence interval
CIN: Cervical intraepithelial neoplasia
CIN1: CIN grade 1
CIN2: CIN grade 2
CIN3: CIN grade 3
CRS: Civil Registration System
DF: Degrees of freedom
DHCS: Danish HIV Cohort Study
DPDB: Danish Pathology Data Bank
HC2: Hybrid capture 2
HPV: Human papillomavirus
HR: High-risk
HrHPV: High-risk human papillomavirus
HSIL: High-grade squamous intraepithelial lesions
LSIL: Low-grade squamous intraepithelial lesions
OR: Odds ratio
PIN: Personal identification number
PLWH: People living with HIV
SHADE: Study on HIV, cervical Abnormalities and infections in women in Denmark
SNOMED: Systemized nomenclature of medicine
WLWH: Women living with HIV

## References

[1] Moscicki AB, Schiffman M, Burchell A, Albero G, Giuliano AR, Goodman MT (2012). Updating the natural history of human papillomavirus and anogenital cancers. Vaccine.

[2] Thomsen LT, Frederiksen K, Munk C, Junge J, Iftner T, Kjaer SK (2015). Long-term risk of cervical intraepithelial neoplasia grade 3 or worse according to high-risk human papillomavirus genotype and semi-quantitative viral load among 33,288 women with normal cervical cytology. Int J Cancer.

[3] Guan P, Howell-Jones R, Li N, Bruni L, de SS FS (2012). Human papillomavirus types in 115,789 HPV-positive women: a meta-analysis from cervical infection to cancer. Int J Cancer.

[4] Clifford GM, Tully S, Franceschi S (2017). Carcinogenicity of human papillomavirus (HPV) types in HIV-positive women: a meta-analysis from HPV infection to cervical Cancer. Clin Infect Dis.

[5] Silver MI, Andrews J, Cooper CK, Gage JC, Gold MA, Khan MJ (2018). Risk of cervical intraepithelial neoplasia 2 or worse by cytology, human papillomavirus 16/18, and colposcopy impression: a systematic review and meta-analysis. Obstet Gynecol.

[6] Markowitz LE, Dunne EF, Saraiya M, Chesson HW, Curtis CR, Gee J (2014). Human papillomavirus vaccination: recommendations of the advisory committee on immunization practices (ACIP). MMWR Recomm Rep.

[7] Lees BF, Erickson BK, Huh WK (2016). Cervical cancer screening: evidence behind the guidelines. Am J Obstet Gynecol.

[8] Ebisch RMF, Ketelaars PJW, van der Sanden WMH, Schmeink CE, Lenselink CH, Siebers AG (2018). Screening for persistent high-risk HPV infections may be a valuable screening method for young women; A retrospective cohort study. PLoS One.

[9] Kelly H, Weiss HA, Benavente Y, de SS MP (2018). Association of antiretroviral therapy with high-risk human papillomavirus, cervical intraepithelial neoplasia, and invasive cervical cancer in women living with HIV: a systematic review and meta-analysis. Lancet HIV.

[10] Denny LA, Franceschi S, de SS HI, Moscicki AB, Palefsky J (2012). Human papillomavirus, human immunodeficiency virus and immunosuppression. Vaccine.

[11] Liu Gui, Sharma Monisha, Tan Nicholas, Barnabas Ruanne V. (2018). HIV-positive women have higher risk of human papilloma virus infection, precancerous lesions, and cervical cancer. AIDS.

[12] Ahdieh Linda, Klein Robert S., Burk Robert, Cu‐Uvin Susan, Schuman Paula, Duerr Ann, Safaeian Mahboobeh, Astemborski Jacquie, Daniel Richard, Shah Keerti (2001). Prevalence, Incidence, and Type‐Specific Persistence of Human Papillomavirus in Human Immunodeficiency Virus (HIV)–Positive and HIV‐Negative Women. The Journal of Infectious Diseases.

[13] Thorsteinsson K, Storgaard M, Katzenstein TL, Ladelund S, Ronsholt FF, Johansen IS (2016). Prevalence and distribution of cervical high-risk human papillomavirus and cytological abnormalities in women living with HIV in Denmark - the SHADE. BMC Cancer.

[14] Adebamowo SN, Olawande O, Famooto A, Dareng EO, Offiong R, Adebamowo CA (2017). Persistent low-risk and high-risk human papillomavirus infections of the uterine cervix in HIV-negative and HIV-positive women. Front Public Health.

[15] de PA KE, de CC MMH, Burchell AN, Klein M (2017). The EVVA Cohort Study: Anal and Cervical Type-Specific Human Papillomavirus Prevalence, Persistence, and Cytologic Findings in Women Living With HIV. J Infect Dis.

[16] Konopnicki D, Manigart Y, Gilles C, Barlow P, de MJ FF (2016). High-risk human papillomavirus genotypes distribution in a cohort of HIV-positive women living in Europe: epidemiological implication for vaccination against human papillomavirus. AIDS.

[17] Brickman C, Palefsky JM (2015). Human papillomavirus in the HIV-infected host: epidemiology and pathogenesis in the antiretroviral era. Curr HIV /AIDS Rep.

[18] Lohse N, Obel N (2016). Update of survival for persons with HIV infection in Denmark. Ann Intern Med.

[19] EACS Guidelines version 9.1 October 2018. Available from: http://www.eacsociety.org/files/2018_guidelines-9.1-english.pdf . Accessed 22 Jan 2019. 23-1-2019. 23-1-2019. Ref Type: Internet Communication.

[20] Kremer WW, Van ZM, Novianti PW, Richter KL, Verlaat W, Snijders PJ (2018). Detection of hypermethylated genes as markers for cervical screening in women living with HIV. J Int AIDS Soc.

[21] Statens Serum Institut. HIV infection and AIDS. http://www.ssi.dk/Service/Sygdomsleksikon/H/AIDS%20-%20HIV.aspx. Accessed 27 April 2017. Webpage in Danish. 27-4-2017. 27-4-2017. Ref Type: Internet Communication.

[22] Obel N, Engsig FN, Rasmussen LD, Larsen MV, Omland LH, Sorensen HT (2009). Cohort profile: the Danish HIV cohort study. Int J Epidemiol.

[23] Thorsteinsson K, Ladelund S, Storgaard M, Ronsholt FF, Johansen IS, Pedersen G (2016). Sexually transmitted infections and use of contraceptives in women living with HIV in Denmark - the SHADE cohort. BMC Infect Dis.

[24] Lauritsen JM & Bruus M. EpiData (version 3.1). A comprehensive tool for validated entry and documentation of data. The EpiData Association, Odense, 2003-2005. 11-2-2015. Ref Type: Generic.

[25] Pedersen CB (2011). The Danish civil registration system. Scand J Public Health.

[26] Erichsen R, Lash TL, Hamilton-Dutoit SJ, Bjerregaard B, Vyberg M, Pedersen L (2010). Existing data sources for clinical epidemiology: the Danish National Pathology Registry and data Bank. Clin Epidemiol.

[27] Solomon D, Davey D, Kurman R, Moriarty A, O'Connor D, Prey M (2002). The 2001 Bethesda system: terminology for reporting results of cervical cytology. JAMA.

[28] Bonde J, Rebolj M, Ejegod DM, Preisler S, Lynge E, Rygaard C (2014). HPV prevalence and genotype distribution in a population-based split-sample study of well-screened women using CLART HPV2 human papillomavirus genotype microarray system. BMC Infect Dis.

[29] Stensen S, Kjaer SK, Jensen SM, Frederiksen K, Junge J, Iftner T (2016). Factors associated with type-specific persistence of high-risk human papillomavirus infection: a population-based study. Int J Cancer.

[30] Meyrelles AR, Siqueira JD, Santos PP, Hofer CB, Luiz RR, Seuanez HN (2016). Bonafide, type-specific human papillomavirus persistence among HIV-positive pregnant women: predictive value for cytological abnormalities, a longitudinal cohort study. Mem Inst Oswaldo Cruz.

[31] Konopnicki D, Manigart Y, Gilles C, Barlow P, de MJ FF (2013). Sustained viral suppression and higher CD4+ T-cell count reduces the risk of persistent cervical high-risk human papillomavirus infection in HIV-positive women. J Infect Dis.

[32] Heard I, Cubie HA, Mesher D, Sasieni P (2013). Characteristics of HPV infection over time in European women who are HIV-1 positive. BJOG.

[33] Tota JE, Bentley J, Blake J, Coutlee F, Duggan MA, Ferenczy A (2017). Approaches for triaging women who test positive for human papillomavirus in cervical cancer screening. Prev Med.

[34] Lorincz AT (2016). Virtues and weaknesses of DNA methylation as a test for cervical Cancer prevention. Acta Cytol.

[35] Thorsteinsson K, Ladelund S, Jensen-Fangel S, Katzenstein TL, Johansen IS, Pedersen G (2016). Incidence of cervical dysplasia and cervical cancer in women living with HIV in Denmark: comparison with the general population. HIV Med.

[36] Strickler HD, Martinson J, Desai S, Xie X, Burk RD, Anastos K (2014). The relation of plasmacytoid dendritic cells (pDCs) and regulatory T-cells (Tregs) with HPV persistence in HIV-infected and HIV-uninfected women. Viral Immunol.

[37] Nygard M, Hansen BT, Dillner J, Munk C, Oddsson K, Tryggvadottir L (2014). Targeting human papillomavirus to reduce the burden of cervical, vulvar and vaginal cancer and pre-invasive neoplasia: establishing the baseline for surveillance. PLoS One.

[38] Woodman CB, Collins SI, Young LS (2007). The natural history of cervical HPV infection: unresolved issues. Nat Rev Cancer.

[39] Jalil EM, Bastos FI, Melli PP, Duarte G, Simoes RT, Yamamoto AY (2013). HPV clearance in postpartum period of HIV-positive and negative women: a prospective follow-up study. BMC Infect Dis.

[40] Branca M, Garbuglia AR, Benedetto A, Cappiello T, Leoncini L, Migliore G (2003). Factors predicting the persistence of genital human papillomavirus infections and PAP smear abnormality in HIV-positive and HIV-negative women during prospective follow-up. Int J STD AIDS.

[41] Dreyer Greta (2018). Clinical implications of the interaction between HPV and HIV infections. Best Practice & Research Clinical Obstetrics & Gynaecology.

[42] Lin Chunqing, Franceschi Silvia, Clifford Gary M (2018). Human papillomavirus types from infection to cancer in the anus, according to sex and HIV status: a systematic review and meta-analysis. The Lancet Infectious Diseases.

[43] Kang M, Cu-Uvin S (2012). Association of HIV viral load and CD4 cell count with human papillomavirus detection and clearance in HIV-infected women initiating highly active antiretroviral therapy. HIV Med.

[44] Lundgren JD, Babiker AG, Gordin F, Emery S, Grund B, Sharma S (2015). Initiation of antiretroviral therapy in early asymptomatic HIV infection. N Engl J Med.

[45] Thorsteinsson K, Ladelund S, Jensen-Fangel S, Johansen IS, Katzenstein TL, Pedersen G (2012). Impact of gender on response to highly active antiretroviral therapy in HIV-1 infected patients: a nationwide population-based cohort study. BMC Infect Dis.

[46] Palefsky JM (2012). Antiretroviral therapy and anal cancer: the good, the bad, and the unknown. Sex Transm Dis.

[47] Fontaine J, Hankins C, Money D, Rachlis A, Pourreaux K, Ferenczy A (2008). Human papillomavirus type 16 (HPV-16) viral load and persistence of HPV-16 infection in women infected or at risk for HIV. J Clin Virol.

[48] Fife KH, Wu JW, Squires KE, Watts DH, Andersen JW, Brown DR (2009). Prevalence and persistence of cervical human papillomavirus infection in HIV-positive women initiating highly active antiretroviral therapy. J Acquir Immune Defic Syndr.

