# Supplementary material for: Persistence of cervical high-risk human papillomavirus in women living with HIV in Denmark – the SHADE
Source: BMC Infect Dis. 2019 Aug 22;19:740. doi: 10.1186/s12879-019-4377-5 (PMC6706931; doi:10.1186/s12879-019-4377-5)
Supplement: Supplementary file 2 — Unadjusted and adjusted odds ratios for predictors of atypical cells of undetermined significance or worse (ASCUS+). A table presenting the unadjusted and adjusted odds ratios for predictors of atypical cells of undetermined significance or worse (ASCUS+). (DOCX 18 kb) [file 12879_2019_4377_MOESM2_ESM.docx]

**Supplementary file 2**

**Unadjusted and adjusted odds ratios for predictors of atypical cells of undetermined significance or worse (ASCUS+) (n = 71)**

| Predictors of persistence | **Normal**  **Cytology**  **(n=44)** | **ASCUS+**    **(n=27)** | **Unadjusted**  **odds ratios** | ***p*-value** | **Adjusted**  **odds ratios^1,^** ^1^ | ***p*-value** | |
| --- | --- | --- | --- | --- | --- | --- | --- |
| **Age when first hrHPV positive^3^, n(%)**  **≥35 years**  **18-34 years**  **(missing)** | 34 (69.4)  10 (45.5)  (0) | 15 (30.6)  12 (54.5)  (0) | 1.00  2.72 (0.97-7.66) | -  0.058 | 1.00  1.74 (0.42-7.27) | -  0.45 | |
| **Race, n(%)**  **White**  **Asian**  Black  (missing)  Combined *p*-value | 21 (63.6)  2 (40.0)  20 (64.5)  (1) | 12 (36.4)  3 (60.0)  11 (35.5)  (1) | 1.00  2.63 (0.38-17.98)  0.96 (0.35-2.67) | -  0.33  0.94  0.58 | 1.00  1.74 (0.18-17.22)  1.07 (0.29-3.96) | -  0.64  0.92  0.90 | |
| ART^4^ duration, (years)  **Median (IQR)**  **(missing)** | 9.4 (4.2-13.3)  (3) | 3.9 (1.1-9.6)  (1) | 0.87 (0.78-0.97) | **0.01** | 0.85 (0.74-0.99) | | **0.03** |
| **AIDS prior to inclusion, n(%)**  **No**  **Yes**  **(missing)** | 36 (66.67)  8 (50.0)  (0) | 18 (33.3)  8 (50.0)  (1) | 1.00  2.00 (0.65-6.20) | -  0.23 | 1.00  3.47 (0.76 -15.81) | | 0.11 |
| **Smoking status, n(%)**  Never smoker  **Current smoker/ Ex-smoker**  **(missing)** | 26 (66.7)  18 (56.3)  (0) | 13 (33.3)  14 (43.7)  (0) | 1.00  1.56 (0.59-4.08) | **-**  0.37 | 1.00  2.40 (0.66-8.78) | | 0.18 |
| **Persistent hrHPV^3^ infection, n(%)**  **No**  **Yes**  **(missing)** | 28 (70.0)  16 (51.6)  (0) | 12 (30.0)  15 (48.4)  (0) | 1.00  2.19 (0.82-5.81) | -  0.12 | 1.00  1.76 (0.54-5.72) | | 0.34 |
| **CD4 count when first hrHPV^3^ positive (cells/μL), n(%)**  **≥350**  **<350**  **(missing)** | 30 (68.2)  9 (56.3)  (5) | 14 (31.8)  7 (43.7)  (4) | 1.00  1.67 (0.52-5.39) | -  0.39 | 1.00  1.69 (0.46-6.14) | | 0.43 |

HrHPV = High-risk human papillomavirus. ART = combined antiretroviral therapy.

^1^The validity of the model was tested using the Hosmer and Lemeshow Goodness-of-Fit Test, ^2^Duration of ART, AIDS prior to inclusion and CD4 count are dependent covariates and where calculated using two models: A model where all variables, but CD4 at inclusion were included and a model where duration of ART and AIDS prior to inclusion were replaced by CD4. We only present the OR of the CD4 count from the second model.
