# Supplementary material for: Persistence of cervical high-risk human papillomavirus in women living with HIV in Denmark – the SHADE
Source: BMC Infect Dis. 2019 Aug 22;19:740. doi: 10.1186/s12879-019-4377-5 (PMC6706931; doi:10.1186/s12879-019-4377-5)
Supplement: Supplementary file 3 — Unadjusted and adjusted odds ratios for predictors of low-grade squamous intraepithelial lesions or worse (LSIL+). A table presenting the unadjusted and adjusted odds ratios for predictors of low-grade squamous intraepithelial lesions or worse (LSIL+). (DOCX 17 kb) [file 12879_2019_4377_MOESM3_ESM.docx]

**Supplementary file 3**

**Unadjusted and adjusted odds ratios for predictors of low-grade squamous intraepithelial lesions or worse (LSIL+) (n = 71)**

| Predictors of persistence | **Normal**  **Cytology**  **(n=45)** | **LSIL+**    **(n=26)** | **Unadjusted**  **odds ratios** | ***p*-value** | **Adjusted**  **odds ratios^1, 2^**  **for LSIL+** | ***p*-value** | |
| --- | --- | --- | --- | --- | --- | --- | --- |
| **Age when first hrHPV positive^3^, n(%)**  **≥35 years**  **18-34 years**  **(missing)** | 35 (71.4)  10 (45.5)  (0) | 14 (28.6)  12 (54.5)  (0) | 1.00  3.00 (1.06-8.51) | **-**  **0.04** | 1.00  2.07 (0.47-9.17) | -  0.34 | |
| **Race, n(%)**  **White**  **Asian**  Black  (missing)  Combined *p*-value | 21 (63.6)  2 (40.0)  21 (67.7)  (1) | 12 (36.4)  3 (60.0)  10 (32.3)  (1) | 1.00  2.62 (0.38 -17.98)  0.83 (0.30-2.35) | -  0.33  0.73  0.51 | 1.00  1.71 (0.16-18.57)  0.93 (0.24-3.67) | -  0.66  0.91  0.89 | |
| ART^4^ duration, (years)  **Median (IQR)**  **(missing)** | 9.4 (4.2-13.3)  (3) | 3.6 (1.1-7.6)  (1) | 0.86 (0.76-0.96) | **0.01** | 0.83 (0.71-0.97) | | **0.02** |
| **AIDS prior to inclusion, n(%)**  **No**  **Yes**  **(missing)** | 37 (68.5)  8 (50.0)  (0) | 17 (31.5)  8 (50.0)  (1) | 1.00  2.18 (0.70-6.78) | -  0.18 | 1.00  4.30 (0.84-21.92) | | 0.08 |
| **Smoking status, n(%)**  Never smoker  **Current smoker/ Ex-smoker**  **(missing)** | 27 (69.2)  18 (56.3)  (0) | 12 (30.8)  14 (43.7)  (0) | 1.00  1.75 (0.66-4.64) | **-**  0.26 | 1.00  2.92 (0.74-11.46) | | 0.12 |
| **Persistent hrHPV^3^ infection, n(%)**  **No**  **Yes**  **(missing)** | 29 (72.5)  16 (51.6)  (0) | 11 (27.5)  15 (48.4)  (0) | 1.00  2.47 (0.92-6.64) | -  0.07 | 1.00  2.11 (0.62-7.22) | | 0.23 |
| **CD4 count when first hrHPV^3^ positive (cells/μL), n(%)**  **≥350**  **<350**  **(missing)** | 31 (70.5)  9 (56.3)  (5) | 13 (29.5)  7 (43.7)  (4) | 1.00  1.86 (0.57-6.04) | -  0.31 | 1.00  1.99 (0.52-7.57) | | 0.31 |

HrHPV = High-risk human papillomavirus. ART = combined antiretroviral therapy.

^1^The validity of the model was tested using the Hosmer and Lemeshow Goodness-of-Fit Test, ^2^Duration of ART, AIDS prior to inclusion and CD4 count are dependent covariates and where calculated using two models: A model where all variables, but CD4 at inclusion were included and a model where duration of ART and AIDS prior to inclusion were replaced by CD4. We only present the OR of the CD4 count from the second model.
