# Supplementary material for: Persistence of cervical high-risk human papillomavirus in women living with HIV in Denmark – the SHADE
Source: BMC Infect Dis. 2019 Aug 22;19:740. doi: 10.1186/s12879-019-4377-5 (PMC6706931; doi:10.1186/s12879-019-4377-5)
Supplement: Supplementary file 4 — Unadjusted and adjusted odds ratios for predictors of high-grade squamous intraepithelial lesions or worse (HSIL+). A table presenting the Unadjusted and adjusted odds ratios for predictors of high-grade squamous intraepithelial lesions or worse (HSIL+). (DOCX 18 kb) [file 12879_2019_4377_MOESM4_ESM.docx]

**Supplementary file 4**

**Unadjusted and adjusted odds ratios for predictors of high-grade squamous intraepithelial lesions or worse (HSIL+) (n = 71)**

| Predictors of persistence | **Normal**  **Cytology**  **(n=63)** | **HSIL+**    **(n=8)** | **Unadjusted**  **odds ratios** | ***p*-value** | **Adjusted**  **odds ratios^1, 2^**  **for HSIL+^4^** | ***p*-value** | |
| --- | --- | --- | --- | --- | --- | --- | --- |
| **Age when first hrHPV positive^3^, n(%)**  **≥35 years**  **18-34 years**  **(missing)** | 44 (89.8)  19 (86.4)  (0) | 5 (10.2)  3 (13.6)  (0) | 1.00  1.39 (0.30-6.41) | -  0.67 | 1.00  2.14 (0.17-26.74) | -  0.56 | |
| **Race, n(%)**  **White**  **Asian**  Black  (missing)  Combined *p*-value | 28 (84.8)  4 (80.0)  29 (93.5)  (2) | 5 (15.2)  1 (20.0)  2 (6.5)  (0) | 1.00  1.40 (0.13-15.26)  0.39 (0.07-2.16) | -  0.78  0.28  0.48 | 1.00  0.64 (0.03-16.56)  0.47 (0.05-4.25) | -  0.79  0.50  0.79 | |
| ART^4^ duration, (years)  **Median (IQR)**  **(missing)** | 7.4 (2.6-12.7)  (4) | 4.8 (2.2-8.1)  (0) | 0.92 (0.79-1.09) | 0.35 | 0.91 (0.73-1.15) | | 0.44 |
| **AIDS prior to inclusion, n(%)**  **No**  **Yes**  **(missing)** | 51 (94.4)  11 (68.7)  (1) | 3 (5.6)  5 (31.3)  (0) | 1.00  7.73 (1.60-37.24) | -  **0.01** | 1.00  8.55 (1.21-60.28) | | **0.03** |
| **Smoking status, n(%)**  Never smoker  **Current smoker/ Ex-smoker**  **(missing)** | 36 (92.3)  27 (84.4)  (0) | 3 (7.7)  5 (15.6)  (0) | 1.00  2.22 (0.49-10.12) | **-**  0.30 | 1.00  2.51 (0.36-17.75) | | 0.36 |
| **Persistent hrHPV^3^ infection, n(%)**  **No**  **Yes**  **(missing)** | 38 (95.0)  25 (80.6)  (0) | 2 (5.0)  6 (19.4)  (0) | 1.00  4.56 (0.85-24.41) | -  0.08 | 1.00  2.78 (0.36-21.49) | | 0.33 |
| **CD4 count when first hrHPV^3^ positive (cells/μL), n(%)**  **≥350**  **<350**  **(missing)** | 39 (88.6)  14 (87.5)  (10) | 5 (11.4)  2 (12.5)  (1) | 1.00  1.11 (0.19-6.41) | -  0.90 | 1.00  1.24 (0.18-8.70) | | 0.83 |

HrHPV = High-risk human papillomavirus. ART = combined antiretroviral therapy.

^1^The validity of the model was tested using the Hosmer and Lemeshow Goodness-of-Fit Test, ^2^Duration of ART, AIDS prior to inclusion and CD4 count are dependent covariates and where calculated using two models: A model where all variables, but CD4 at inclusion were included and a model where duration of ART and AIDS prior to inclusion were replaced by CD4. We only present the OR of the CD4 count from the second model.
